# Prevalence and Diversity of Antibiotic Resistance Genes in Swedish Aquatic Environments Impacted by Household and Hospital Wastewater

**DOI:** 10.3389/fmicb.2019.00688

**Published:** 2019-04-04

**Authors:** Faisal Ahmad Khan, Bo Söderquist, Jana Jass

**Affiliations:** ^1^The Life Science Centre – Biology, School of Science and Technology, Örebro University, Örebro, Sweden; ^2^School of Medical Sciences, Faculty of Medicine and Health, Örebro University, Örebro, Sweden

**Keywords:** carbapenemase, urban wastewater, surface water, enterobacteriaceae, VIM-1, extended-spectrum beta-lactamase, antimicrobial resistance gene co-occurrence

## Abstract

Antibiotic-resistant Enterobacteriaceae and non-lactose fermenting Gram-negative bacteria are a major cause of nosocomial infections. Antibiotic misuse has fueled the worldwide spread of resistant bacteria and the genes responsible for antibiotic resistance (ARGs). There is evidence that ARGs are ubiquitous in non-clinical environments, especially those affected by anthropogenic activity. However, the emergence and primary sources of ARGs in the environment of countries with strict regulations for antibiotics usage are not fully explored. The aim of the present study was to evaluate the repertoire of ARGs of culturable Gram-negative bacteria from directionally connected sites from the hospital to the wastewater treatment plant (WWTP), and downstream aquatic environments in central Sweden. The ARGs were detected from genomic DNA isolated from a population of selectively cultured coliform and Gram-negative bacteria using qPCR. The results show that hospital wastewater was a reservoir of several class B β-lactamase genes such as *bla*_IMP-1_*, bla*_IMP-2_, and *bla*_OXA-23_, however, most of these genes were not observed in downstream locations. Moreover, β-lactamase genes such as *bla*_OXA-48_, *bla*_CTX-M-8_, and *bla*_SFC-1_, *bla*_V IM-1_, and *bla*_V IM-13_ were detected in downstream river water but not in the WWTP. The results indicate that the WWTP and hospital wastewaters were reservoirs of most ARGs and contribute to the diversity of ARGs in associated natural environments. However, this study suggests that other factors may also have minor contributions to the prevalence and diversity of ARGs in natural environments.

## Introduction

Antibiotic resistance is a serious human health problem that jeopardizes the efficacy of the current antibiotic treatment for life-threatening bacterial infections (Hawkey and Jones, 2009). It is estimated that over 25,000 people die in Europe each year due to infections caused by antibiotic-resistant bacteria (Borg, 2012). A major reservoir of antibiotic-resistant Gram-negative bacteria is the gut microbiota of human and animals, especially after exposure to antibiotics. Most antibiotic resistance genes (ARGs) are found on mobile genetic elements, such as plasmids, transposons, and integrons that can be transferred between different bacterial species through horizontal gene transfer (HGT) (Bahl et al., 2004; Goren et al., 2010). As a result of the excessive use of antimicrobial compounds for clinical and agricultural purposes, antibiotic-resistant bacteria and ARGs have become widespread in natural environments (Baquero et al., 2008). The occurrence of ARGs in non-clinical environments has been highlighted by several recent studies (Spindler et al., 2012; Khan et al., 2013; Rutgersson et al., 2014; Xiong et al., 2014; Rodriguez-Mozaz et al., 2015). The majority of these studies have focused on highly contaminated environments associated with the pharmaceutical industry and other anthropogenic activities. Furthermore, epidemiology of antibiotic resistance has largely been limited to clinical settings, however, it is becoming clear that natural environments should be monitored for antibiotic resistance for a more comprehensive understanding of the prevalence, distribution, and transmission of ARGs (Wright, 2010).

According to the European Antimicrobial Resistance Surveillance Network (EARS-Net), clinical reports of antibiotic-resistant Gram-negative bacteria are increasing. In general, the Scandinavian countries exhibit low percentages of antibiotic resistance, while these levels are higher in Southern and Eastern European countries (ECDC, 2012). This variation in resistance is likely related to differences in policies and strategies of antibiotic usage in these countries. It is proposed that the effluents from municipal wastewater treatment plants (WWTP) are hotspots for the emergence of clinically relevant Gram-negative pathogens and ARGs and their spread into the environment (Marti et al., 2013; Hembach et al., 2017). The diverse population of bacteria from the household, hospital and industrial wastewaters reach the WWTP, where conditions for HGT are optimal due to the formation of biofilms and stress caused by various pharmaceutical and heavy metal contamination (Karkman et al., 2018). Since the use of antibiotics in clinical environments select for antibiotic-resistant bacteria, there is concern that effluents from hospitals and WWTPs provide a continuous source of ARGs in the environment (Livermore, 2005). A recent study reported the dispersion of bacteria and ARGs from a WWTP into downstream aquatic environments using 2-dimensional hydraulic simulations (Jäger et al., 2018). Moreover, hospitals are not the only source of antibiotic resistance in the environment. According to the European Centre for Disease Prevention and Control, a significant proportion of antibiotics consumed by humans is in the community rather than in healthcare settings (ECDC, 2012; Folkhälsomyndigheten, 2016), and a major part of the antibiotics taken by humans and animals are excreted unaltered in urine and feces (Uyaguari-Díaz et al., 2018). Thus, antibiotics used in outpatient therapy are also released into the wastewater systems and likely contribute to the selection pressure and enrichment of ARGs in WWTP.

Antibiotic usage in Sweden is continually decreasing due to the regulations for use in both medicine and agriculture, however, reports of infections with antibiotic-resistant Enterobacteriaceae are still increasing (Folkhälsomyndigheten, 2016). However, in 2016, more than 70% of patients in Sweden with carbapenemase-producing Enterobacteriaceae were infected abroad (Folkhälsomyndigheten, 2016). It was previously shown that a low-level presence of carbapenemase-producing Enterobacteriaceae in a Swedish river was likely transmitted from a nearby hospital (Khan et al., 2018). In environments with low pharmaceutical contamination, the presence of ARGs are more closely linked to coliform and fecal contamination (Karkman et al., 2019). Thus the aim of the present study was to investigate the presence and diversity of ARGs of culturable Gram-negative bacteria from the hospital, and municipal wastewater, recipient river, and lake in central Sweden. This was based on the hypothesis that diverse clinically relevant ARGs are already present in the environment but are not detected due to their low-level presence.

## Materials and Methods

### Sampling Sites

**FIGURE 1:**
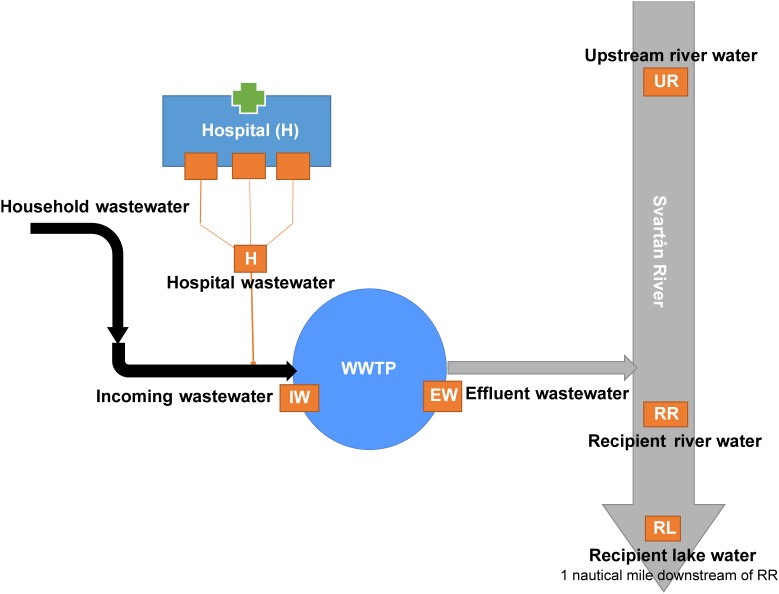
Illustration of the sample sites along the wastewater system in Örebro city, Sweden. Wastewater from households, industries, and the hospital are transported to the wastewater treatment plant (WWTP) located along the Svartån river downstream of Örebro City. Svartån River flows through the city and exits into Hjälmaren Lake. Sample locations are indicated as orange boxes. Wastewater samples were collected from hospital effluent (H), and incoming wastewater (IW), and treated effluent wastewater (EW) at the WWTP. Environmental water samples were collected from Svartån River upstream of Örebro city (UR), Svartån River downstream of WWTP (RR) and Hjälmaren Lake (RL), which is one nautical mile downstream of RR.

The study was performed in Örebro, a city located in central Sweden with a population of 143,000 inhabitants ^1^. It has one hospital that serves as a referral hospital for approximately 270,000 inhabitants from Örebro city and surrounding municipalities. A schematic illustration of the sampling sites and how they are connected is shown in Figure 1. The effluent wastewater from the different hospital buildings is transported through the drainage system into WWTP without prior treatment. The hospital wastewater joins the community and industrial wastewater collection system that leads to the WWTP. The Örebro WWTP processes approximately 45,000 m^3^ of wastewater per day (see footnote 1). Upon entering the WWTP, the wastewater undergoes mechanical treatment to remove solid waste before it enters the aeration tanks for biological treatment. The solid waste and the flocculated active sludge from the sedimentation basins are processed in mesophilic digesters and the resulting leachates are returned to the aeration tanks. Finally, the wastewater goes through a chemical treatment to remove phosphorus using iron sulfate precipitation before it is discharged. The treated wastewater is discharged into the river and the downstream Hjälmaren Lake. In the present study, samples were collected from the hospital wastewater (H) and the incoming and effluent waters at the WWTP. The incoming wastewater (IW) was collected after mechanical treatment and the effluent (EW) wastewater before it was released into the river from the WWTP. The environmental waters were collected from Svartån River, upstream of Örebro city (UR) and downstream of the treated wastewater effluent (referred to as recipient river water, RR) and the recipient Hjälmaren Lake, which receives the water from Svarån River (referred to as recipient lake water, RL).

### Sample Collection and Processing

Three independent samples were collected from each site in 1-liter sterile borosilicate glass bottles during June 2016 and stored at 6°C prior to processing within 24 h. Surface water samples (approximate depth 0.5 m) were collected from three points over 100 m^2^ area across the flow of the water from Svartån river (UR and RR) and the recipient Hjälmaren lake (RL). Samples from the WWTP (IW, EW) were collected three times within a 12 h period. The hospital wastewater was collected from the outlet pipes of three main buildings (A-, B-, and M-house) where the departments of infectious diseases, surgery, pediatrics, urology, and medicine are located. To enrich the culturable coliforms and other Gram-negative bacteria present at low levels, 100 mL of water from each sample replicate was filtered through 0.45 μm polyethylene sulfonate membrane filters (Sartorius Stedim Biotech, Sweden) and placed onto Chromocult coliform agar (Merck, Darmstadt, Germany) plates. The agar plates were incubated at 37°C to obtain confluent bacterial growth. After 24 h of incubation, the filter paper was transferred to a 15 mL sterile falcon tube containing 10 mL phosphate-buffered saline (PBS) and vortexed to collect the cultures from filter paper. The bacterial suspension was transferred to a new 15 mL falcon tube, centrifuged at 4,800 ×*g* for 5 min to obtain a bacterial pellet and the pellet was stored in -80°C for DNA extraction.

The coliform enumeration was performed for each sample by filtering appropriate volumes through 0.45 μm polyethylene sulfonate membrane filters (Sartorius Stedim Biotech, Sweden) and cultivating on Chromocult coliform agar plates. Colonies were counted after 24 h of growth at 37°C and cfu/100 mL were calculated.

### DNA Extraction

DNA from the mixed population of culturable coliforms and other Gram-negative bacteria growing on Chromocult coliform agar was isolated using the guanidinium thiocyanate-phenol-chloroform method described previously (Lemarchand et al., 2005). Briefly, the pellet was dissolved in Tris–EDTA buffer and lysed in GES lysis buffer (500 mM guanidium thiocyanate, 100 mM EDTA, 0.5% (w/v) *n*-lauryl-sarcosine). The DNA was extracted by adding ammonium acetate (4 M, pH 5.2) and phenol:chloroform:isoamyl alcohol (25:24:1, pH 8.0). DNA was precipitated with ice-cold isopropanol and washed with 70% ethanol. After washing, the DNA pellet was dried at 40°C and dissolved in nuclease-free water. DNA quantification was performed using the DS-11 spectrophotometer (DeNovix Inc., Wilmington, DE, United States).

### Detection of Bacterial Species Cultured on Chromocult Coliform Agar

Chromocult coliform agar was used for the enrichment of total coliforms, including *Escherichia coli*, and other Gram-negative bacteria from environmental samples (Finney et al., 2003). To determine the selectivity of the Chromocult coliform agar, and to confirm the growth of important human pathogens, four samples representing different environments including hospital wastewater, WWTP wastewater (combined incoming and effluent water), upstream in the river and downstream of the WWTP (combined downstream river and lake water) were tested for the presence of 116 microbial species. The DNA isolated from the mixed bacterial population growing on Chromocult coliform agar were tested for the presence of bacterial and fungal species using a Microbial DNA qPCR array (Qiagen, Sweden. BAID-1405ZRA, BAID-1903ZRA). The microbial species included in the array are listed in the Supplementary Table S1. Each assay was based on PCR amplification of a species-specific region of 16S rRNA gene. The array included two pan-bacteria primer-sets that serve as controls for the presence of bacterial DNA by amplifying the evolutionarily conserved regions of the 16S rRNA gene. The positive PCR control included in the array tests for the presence of PCR inhibitors and the qPCR efficiency. For microbial identification, samples from incoming (IW) and effluent water (EW) were combined to get a representative sample for the WWTP. Both recipient river (RR) and lake water (RL) samples were also combined.

The qPCR was performed on a Stratagene Mx3000P thermocycler (Agilent Technologies, United States) in a 25 μL reaction containing 12.75 μL of the qPCR master mix (DNA polymerase and ROX passive reference dye) and 10 ng of template DNA in milli-Q water. PCR cycling conditions were: initial PCR amplification at 95°C for 10 min, followed by 40 cycles of denaturation at 95°C for 15 s and the annealing/extension at 60°C for 2 min. Fluorescence for FAM (6-carboxyfluorescein) was recorded during the annealing/extension step. The *C*_T_ values for all qPCR runs were calculated with a threshold value of 0.1, as recommended by the manufacturer. A no-template control (NTC) was performed as a baseline for gene identification and *C*_T_ values were exported to Data Analysis Template Excel Software using the ΔC_T_ method, Δ*C_T_* = *C_T-testsample_*-*C_T-notemplatecontrol_*. Gene identification was determined as positive when Δ*C*_T_ ≥ 6, negative when Δ*C*_T_ < 3, and inconclusive when Δ*C*_T_ ≥ 3. For inconclusive identification, the *C*_T_ value below 34 was considered positive identification, while the *C*_T_ value above 34 was considered as no product.

### Detection of Antibiotic Resistance Genes by High-Throughput qPCR

High-throughput detection of ARGs was performed using ARGs DNA qPCR Array (Qiagen, Sweden. BAID-1901ZRA), following the manufacturer’s instructions. The 96-well array simultaneously detects 84 ARGs (including allelic variants) belonging to different antibiotic classes; aminoglycosides, class A, B, C, and D β-lactamases, fluoroquinolones, macrolide, lincosamide, streptogramin B, tetracycline, vancomycin, and multidrug resistance (Supplementary Table S2). The samples from hospital wastewater (H), incoming (IW) and effluent (EW) wastewater from WWTP, recipient river (RR) and lake (RL) water, and upstream river (UR) were tested for the presence of ARGs. Gene identification was performed using the Δ*C*_T_ method as described above.

### Statistical Analysis

Student’s *t-*test (un-paired, two-tailed) was used to compare coliform numbers from IW and effluent wastewater (EW) from WWTP. Non-metric Multidimensional Scaling (NMDS) was applied to ARGs presence-absence data using the correlation matrix in the PAST software package (Version 3.14) by Palaeontologica Electronica (Hammer et al., 2001). To visualize the co-occurrence of ARGs, a correlation matrix was constructed using pairwise Spearman’s correlation between ARGs detected in ≥ 6 out of 18 samples (including the biological replicates). To reduce the false-positive associations, a high Spearman’s correlation coefficient (*p*) of ≥ 0.8 and *P*-value of < 0.01 was considered as the statistically reliable association between two ARGs. The co-occurrence network was constructed and visualized in Gephi using Fruchterman Reingold layout (Bastian et al., 2009). The co-occurrence network was further classified into sub-networks (modules) based on their modularity class (Vincent et al., 2008). The Venn diagram was constructed using InteractiVenn (Heberle et al., 2015) for three sample sources: hospital wastewater, WWTP (both incoming and outgoing wastewater), and environmental water (upstream river and downstream river and lake water). A gene detected in any of the three samples from one sampling site was included in the analysis.

## Results

### Growth of Diverse Bacterial Species on Chromocult Coliform Agar

The study was performed in Örebro city located in central Sweden and is a representative of most Nordic cities in terms of population density, tourism, environmental impact, and wastewater management. The city WWTP receives wastewater from households, local industries, and a hospital. After wastewater treatment, the effluent is discharged into Svartån River that flows through the city and discharges downstream into Hjälmaren Lake. The six sample sites selected for the study include hospital wastewater, incoming wastewater, and outgoing treated water at the WWTP, upstream and downstream in the Svartån river and Hjälmaren lake (Figure 1).

To determine the growth of important human pathogens on Chromocult coliform agar, DNA was extracted from the bacterial growth from the hospital wastewater, WWTP wastewater, and environmental waters and were analyzed for the presence of 116 bacterial and fungal species. Bacteria belonging to four phyla: *Proteobacteria, Firmicutes, Actinobacteria*, and *Bacteroidetes* were present among different samples (Table 1). The common Gram-negative pathogens such as *E. coli, Klebsiella oxytoca, Aeromonas* spp. and *Citrobacter freundii* were present in all four environments. However, *Acinetobacter baumanii* was detected only in the WWTP and recipient river and lake water. *Pseudomonas aeruginosa* was present in all samples except in the upstream river water. Most of the species belonging to *Actinobacteria* were identified in the hospital wastewater but were not detected in the WWTP and recipient waters. The greatest number of species was identified in the effluent wastewater from the WWTP (26 species), while the upstream river water had the least number of species (18 species). *Proteobacteria* was prominent in all samples, making up to 78% of the total identified community in all the samples. The remaining community consisted of 15% *Firmicutes*, 7% *Actinobacteria* and 1% *Bacteroidetes.*

**Table 1:** Bacterial species in water samples from the hospital, wastewater treatment plant, and environmental waters detected using DNA qPCR array.

	Hospital	WWTP	Receiving water	Upstream river
***Proteobacteria***				
*Achromobacter xylosoxidans*	+	+	+	
*Acinetobacter baumannii*		+	+	
*Acinetobacter calcoaceticus^∗^*	+	+	+	+
*Aeromonas* spp.	+	+	+	+
*Alcaligenes faecalis*	+	+	+	+
*Arcobacter butzleri*	+			
*Brevundimonas diminuta*	+		+	
*Brevundimonas vesicularis^∗^*	+		+	
*Burkholderia* spp.	+	+	+	
*Campylobacter upsaliensis*				+
*Citrobacter freundii*	+	+	+	+
*Escherichia coli^∗^*	+	+	+	+
*Ewingella americana^∗^*		+	+	+
*Hafnia alvei*	+	+	+	+
*Helicobacter pylori*				+
*Klebsiella oxytoca^∗^*	+	+	+	+
*Morganella morganii*	+	+	+	+
*Ochrobactrum* spp.	+			
*Pantoea* spp.		+	+	+
*Plesiomonas shigelloides*			+	+
*Proteus* spp.	+	+	+	
*Pseudomonas aeruginosa*	+	+	+	
*Salmonella enterica*		+	+	
*Vibrio cholerae*			+	
*Xanthomonas retroflexus^∗^*	+	+	+	
*Yersinia enterocolitica*		+	+	
***Actinobacteria***				
*Actinomyces gerencseriae*	+			+
*Actinomyces israelii*	+			+
*Brevibacterium casei*	+			
*Micrococcus luteus*	+			
***Firmicutes***				
*Bacillus* spp.	+	+	+	+
*Enterococcus faecalis*	+	+	+	
*Enterococcus faecium*	+	+	+	
*Enterococcus gallinarum^∗^*		+	+	
*Lactobacillus gasseri*				+
*Ruminococcus bromii*	+			+
***Bacteroidetes***				
*Bacteroides vulgatus*		+		


*^∗^Possibility of presence of other related species. See Supplementary Table S1.*

Although Chromocult coliform agar is a selective media for Gram-negative bacteria, some Gram-positive species from the phylum *Firmicutes* and *Actinobacteria* were still able to grow, such as *Bacillus* spp., *Enterococcus* spp., *Actinomyces israelii, Brevibacterium casei*, and *Micrococcus luteus* (Table 1). However, *C*_T_ values for *Enterococcus* spp. were high (above 30 in most samples), which indicates that they were detected at very low levels likely due to poor or no growth on Chromocult coliform agar. *Bacteroides vulgatus* was identified in WWTP, which was the only member of *Bacteroidetes* identified in all the samples. There were no fungal species detected from any samples grown on Chromocult coliform agar.

**FIGURE 2:**
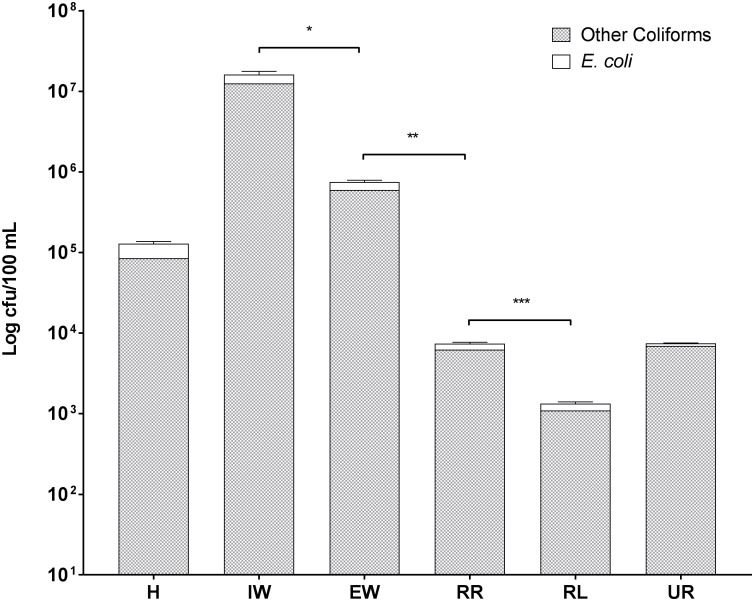
Levels of *E. coli* and other coliform bacteria in wastewaters and recipient environmental waters in Örebro Sweden. Symbols in the figure represent the following: H, hospital wastewater; IW, incoming sewage water from the wastewater treatment plant; EW, effluent wastewater from the wastewater treatment plant; UR, surface water from Svartån river upstream of Örebro city; RR, surface water from Svartån river water downstream of wastewater treatment plant; and RL, surface water from Hjälmaren lake downstream of wastewater treatment plant. Statistical significance is shown by ^∗^ for *P* ≤ 0.05, ^∗∗^ for *P* ≤ 0.01, and ^∗∗∗^ for *P* ≤ 0.001 as determined by Student’s *t-*test. Error bars represent the standard deviation of three biological replicates (*n* = 3).

Enterobacteriaceae were present in all six sites, including in the river upstream of the city (Figure 2). Total coliform counts including *E. coli* were highest in the wastewater coming into the WWTP with 1.6 × 10^7^ cfu/100 mL, while the lowest levels were found in the recipient lake water with 1.3 × 10^3^ cfu/100 mL. It is interesting that similar levels of total coliforms including *E. coli* were observed in samples from the river upstream of Örebro city (UR) and downstream of the WWTP discharge (RR), having 7.5 × 10^3^ cfu/100 mL and 7.4 × 10^3^ cfu/100 mL, respectively. However, the numbers of *E. coli* and other coliforms were significantly reduced in downstream lake water (Figure 2). The outgoing sewage water showed a significantly decreased abundance of *E. coli* and other coliforms compared to incoming sewage water (from 3.6 × 10^6^ to 1.5 × 10^5^ cfu/100 mL for *E. coli*, from 1.26 × 10^7^ to 6 × 10^6^ cfu/100 mL for other coliforms). *E. coli* numbers in recipient river water were 1.1 × 10^3^ cfu/100 mL, making it unsafe for bathing according to European bathing water quality directive (EU, 2006).

### Antibiotic Resistance Genes in Wastewater and Environmental Samples

**FIGURE 3:**
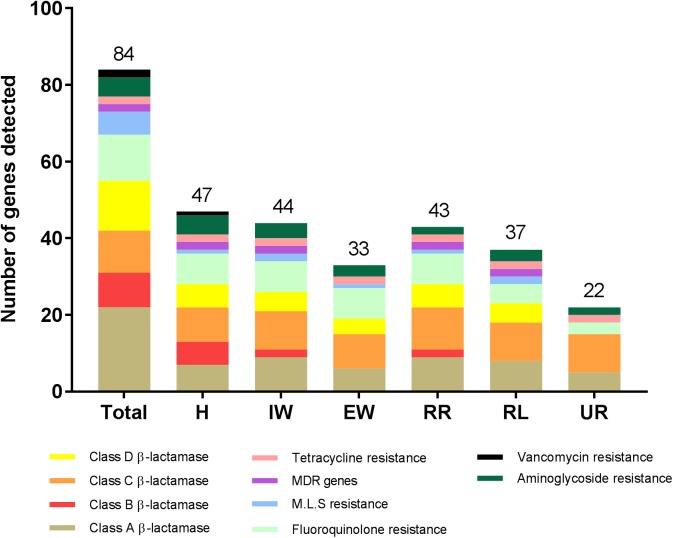
The number of antibiotic resistance genes (ARGs) detected from wastewater and environmental waters in Örebro Sweden. Wastewater samples were taken from hospital effluent (H), incoming wastewater to WWTP (IW), and effluent wastewater (EW). Environmental water samples were taken from Svartån River upstream of Örebro city (UR), Svartån River downstream of WWTP (RR) and Hjälmaren Lake (HF), which is one nautical mile downstream of RR. The number at the top of each bar indicates the total number of detected ARGs in a particular location. MDR indicates multi-drug resistance efflux pump genes. M.L.S indicates genes conferring resistance to either of the macrolide, lincosamide, and streptogramin B antibiotics.

Wastewater from the hospital, WWTP and environmental water samples were analyzed for the presence of 84 ARGs conferring resistance to all major classes of antibiotics (Supplementary Table S2). Altogether, 55 out of 84 ARGs were detected in all the sites (Tables 2, 3). Out of the 55 ARGs, 18 (33%) were detected at all sites and can be considered as widespread. These include genes belong to class A β-lactamase (*bla*_CTX-M-1_*, bla*_SHV -156G_*, bla*_SHV -238G240E_), class C β-lactamase (*bla*_ACT-1_*, bla*_ACT-5/7_*, bla*_CMY -10_*, bla*_DHA_*, bla*_FOX_*, bla*_LAT_*, bla*_MIR_*, bla*_MOX_), fluoroquinolones resistance (*qnrB-1, qnrB-5, qnrB-8*), tetracycline resistance (*tetA*, *tetB*), and aminoglycosides resistance (*aacC2*, *aadA1*). The highest number of ARGs was observed in samples from hospital wastewater (47 out of 84), while the lowest number was found in water samples from the upstream river (22 out of 84) before the WWTP effluent (Figure 3). Class B β-lactamases are clinically important carbapenemases and the majority of this class included in this study were present in hospital wastewater (*bla*_IMP-1_*, bla*_IMP2_*, bla*_IMP-5_*, bla*_IMP-12_*, bla*_V IM-1_, and *bla*_V IM-13_).

**Table 2:** β-lactamase genes identified in samples from hospital wastewater, WWTP, river, and lake water in Sweden.

Gene name/classification	Hospital	WWTP IN	WWTP OUT	Recipient river	Recipient lake	Upstream river
**Class A β-lactamase^1^**		
*bla*_CTX-M-1_ group	+++	+++	+++	+++	+++	+++
*bla*_CTX-M-8_ group				++		
*bla*_CTX-M-9_ group	+++	+++	+++	+++	+++	
*bla*_GES_	+++	+++				
*bla*_Per-1_ group		++	+	+		
*bla*_SFC-1_					+	
*bla*_SFO-1_	+++	++		+++	++	+++
*bla*_SHV_	+++	++	+++	+++	++	+++
*bla*_SHV (156G)_	+++	++	+++	+++	+++	+++
*bla*_SHV (238G240E)_	+++	++	+++	+++	+++	+++
*bla*_V EB_		+++		+++	+	
**Class B β-lactamase^2^**		
*bla*_IMP-1_ group	++					
*bla*_IMP-12_ group	+++	+				
*bla*_IMP-2_ group	+++					
*bla*_IMP-5_ group	+++	+				
*bla*_V IM-1_ group	+++			++		
*bla*_V IM-13_	+++			++		
**Class C β-lactamase**		
*bla*_ACC-1_ group	+++	+++		+++	+++	+++
*bla*_ACC-3_			+++	+++	+++	+++
*bla*_ACT-5/7_ group	+++	+++	+++	+++	+++	+++
*bla*_ACT-1_ group	+++	+++	+++	+++	+++	+++
*bla*_CFE-1_		++		+		
*bla*_CMY -10_ group	+++	+++	+++	+++	+++	+++
*bla*_DHA_	+++	+++	+++	+++	+++	+++
*bla*_FOX_	+++	+++	+++	+++	+++	+++
*bla*_LAT_	+++	+++	+++	+++	+++	+++
*bla*_MIR_	+++	+++	+++	+++	+++	+++
*bla*_MOX_	+++	+++	+++	+++	+++	+++
**Class D β-lactamase^3^**		
*bla*_OXA-10_ group	+++	+++	+++	+++	+++	
*bla*_OXA-2_ group	+++	+++	+++	+++	+++	
*bla*_OXA-23_ group	++					
*bla*_OXA-48_ group				+++	++	
*bla*_OXA-50_ group	+++	+++	+++	+++	++	
*bla*_OXA-51_ group	+++	+++	+++	+++	++	
*bla*_OXA-58_ group	+++	+++		+		


*+ detected in one replicate, ++ detected in two replicates, +++ detected in three replicates. The* β*-lactamase genes that were not detected from any sample; ^1^(bla_BES-1_, bla_BIC-1_, bla_IMI_, bla_NMC-A_, bla_KPC_, bla_Per-2_ group, bla_SHV(156D)_, bla_SHV(156G)_, bla_SHV(238G240K)_, bla_SHV(238S240E)_, bla_SHV(238S240K)_, bla_SME_, bla_TLA-1_); ^2^(bla_ccrA_, bla_NDM_, bla_VIM-7_); ^3^(bla_OXA-18_, bla_OXA-24_, bla_OXA-45_, bla_OXA-54_, bla_OXA-55_).*

**Table 3:** Antibiotic resistance genes (other than β-lactamase genes) identified in samples from hospital wastewater, WWTP, river and lake water in Sweden.

Gene name/classification	Hospital	WWTP IN	WWTP OUT	Recipient river	Recipient lake	Upstream river
**Fluoroquinolone resistance^1^**						
*qnrA*	+++	+	++	+		
*qnrB-1* group	+++	+++	+++	+++	+++	++
*qnrB-4* group	++	++	+++	+++		
*qnrB-5* group	+++	+++	+++	+++	+++	+++
*qnrB-8* group	+++	+++	+++	+++	+++	+++
*qnrD*	+++	+++	+++	+++		
*qnrS*	+++	+++	+++	+++	+++	
*aac(6)-Ib-cr*	+++	+++	+++	+++	+++	
**Macrolide resistance^2^**						
*mefA*		++			+	
**Macrolide-Lincosamide-Streptogramin B resistance^3^**						
*ermB*	+++	+++	+++	+++	+	
**Multidrug efflux pump**						
*oprJ*	+++	+++		+++	+	
*oprM*	+++	+++		+++	+	
**Tetracycline resistance**						
*tetA*	+++	+++	+++	+++	+++	+++
*tetB*	+++	+++	+++	+++	+++	+++
**Aminoglycoside resistance**						
*aacC1*	+++	+				
*aacC2*	+++	++	+++	+++	+++	++
*aacC4*	+	+	+		+	
*aadA1*	+++	+++	+++	+++	+++	+
*aphA6*	+++					
**Vancomycin resistance^4^**						
*vanC*	+++					


*+ detected in one replicate, ++ detected in two replicates, +++ detected in three replicates. Antibiotic resistance genes that were not detected from any sample; ^*1*^*qepA, qnrB-31 and qnrC*; ^*2*^*ereB*; ^*3*^*ermA, ermC*; ^*4*^*vanB*; and msrA.*

**FIGURE 4:**
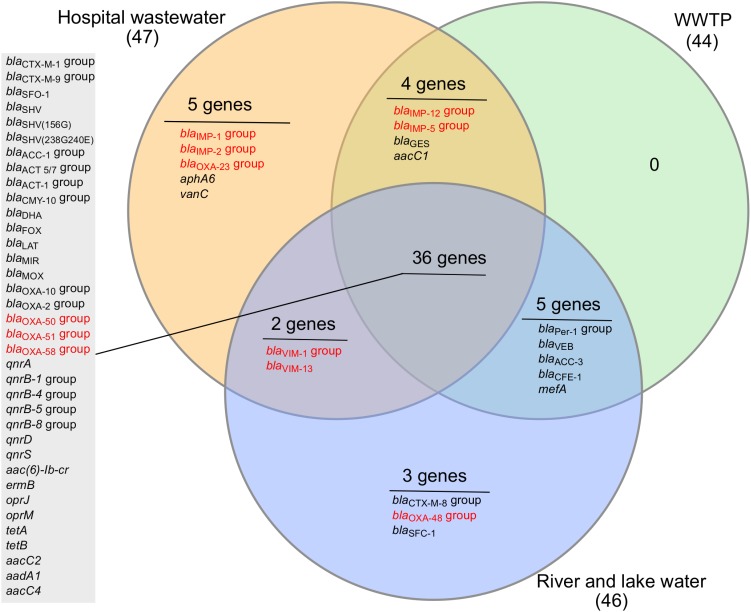
Venn diagram of antibiotic-resistance genes detected in the hospital wastewater, wastewater treatment plant (WWTP), and recipient river and lake waters. The number in the bracket indicates the total number of genes detected from a particular sample type. The results from all six samples were divided into three sets based on the sample type; hospital wastewater, wastewater from WWTP (combined incoming and effluent wastewater), and aquatic environments (upstream river and downstream river and lake water). The genes detected in all sample types are listed in the gray-shaded box. Carbapenemase genes are highlighted in red color.

Thirty-six ARGs were commonly present in all three environments (hospital wastewater, WWTP wastewater, and recipient aquatic environments) and predominantly belonging to Class A-, C-, D- β-lactamases, and quinolone resistance genes (Figure 4). There were no unique genes detected in the WWTP. The hospital wastewater appeared to be a reservoir of diverse ARGs as a higher number of unique ARGs were observed (Figure 4). These include carbapenemase genes *bla*_IMP-1_*, bla*_IMP-2_*, bla*_OXA-23_, aminoglycoside resistance gene *aphA*, and vancomycin resistance gene *vanC*. The *vanC* is a chromosomally located gene generally associated with the Gram-positive *Enterococcus gallinarum* and was recently detected in *Enterococcus faecium* (Sun et al., 2014). Since both *E. faecium* and *Enterococcus faecalis* were detected on Chromocult coliform agar, the presence of *vanC* in hospital wastewater is not unexpected. Although *E. faecium* was present in all the samples tested except upstream river water, *vanC* was detected only in hospital wastewater.

The number of ARGs were reduced considerably in effluent water from WWTP (33 out of 84), compared to incoming sewage water (44 out of 84). However, the number of ARGs found in the receiving river water was higher (43 out of 84) than effluent water from WWTP (Figure 3). The carbapenemase genes, *bla*_V IM-1_ and *bla*_V IM-13_ present in hospital wastewater were not detected downstream in WWTP. However, these genes were detected in the downstream river receiving effluent water from WWTP (Table 2 and Figure 4). Moreover, *bla*_OXA-48_ was found in the recipient river and lake waters but was not detected in other locations (Table 2).

### Similarity Between ARG Profiles in Environmental Samples

**FIGURE 5:**
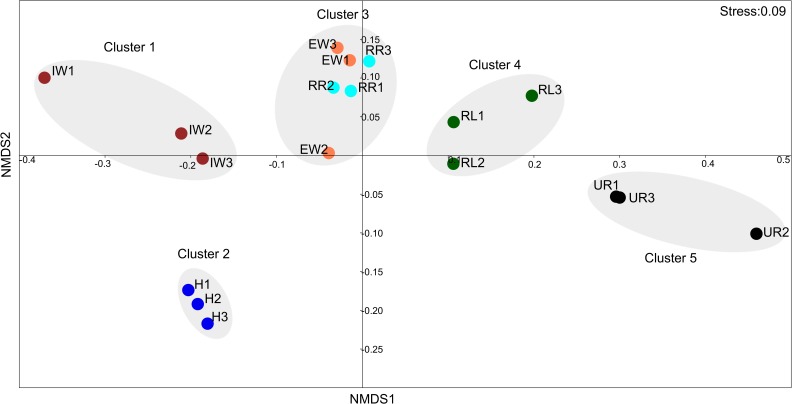
Non-metric multidimensional scaling plot displaying similarities between ARG profiles of hospital wastewater, WWTP wastewater, and environmental water samples. Three replicate samples taken from each location are represented by 1, 2, and 3 after the symbol. Symbols in the figure represent the following: H, Hospital wastewater; IW, Incoming sewage water from the wastewater treatment plant; EW, effluent wastewater from the wastewater treatment plant; UR, surface water from Svartån river upstream of Örebro city; RR, surface water from Svartån river water downstream of the wastewater treatment plant; and RL, surface water from Hjälmaren lake downstream of the wastewater treatment plant. A low-stress value (0.09) indicates a good quality ordination.

The relationship between the ARG profiles of different samples was evaluated by NMDS. The NMDS plot showed five distinct clusters of samples from the hospital, incoming sewage water, effluent water from the WWTP and receiving river water, receiving lake water, and upstream river water (Figure 5). The samples from the same source generally grouped together, however, the samples collected from WWTP effluent (EW) and receiving river water (RR) were related to each other and are grouped together (cluster 3). Water samples from the upstream river contained fewer ARGs compared to the rest of the samples, thus forming a cluster in a separate quadrant (cluster 5). Although hospital wastewater (H) and downstream wastewater (IW) were clustered in one half of the plot due to the presence of a high number of ARGs, however, they were grouped separately.

### Co-occurrence of ARGs in Environmental Samples

**FIGURE 6:**
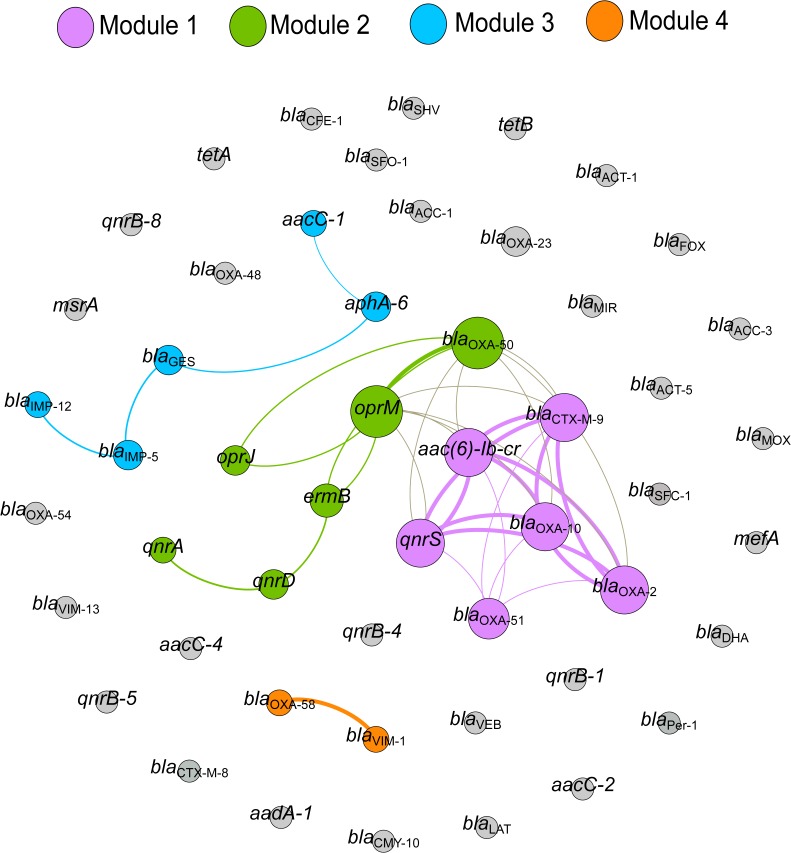
Network plot showing co-occurrence of ARGs detected in wastewater and environmental water samples in Örebro, Sweden. The co-occurrence network is based on a correlation matrix among detected ARGs using Spearman’s rank correlation. Only correlations with a high correlation coefficient (*p ≥* 0.8) and low *P*-value (*P-*value 0.01) are shown. The node size represents the number of connections with other ARGs. The thickness of the edge is proportional to the Spearman’s correlation coefficient (*p*) of the connection. The co-occurrence network is further divided into sub-networks (modules) based on modularity class (modularity index = 0.477). Genes in gray color do not show a significant correlation.

The co-occurrence relationship among ARGs was explored using Spearman’s correlation. The network consists of 51 nodes (ARGs) and 37 edges (significant connections) (Figure 6). Based on the modularity class, the network can be divided into four sub-networks (modules). Module 1 consists of six ARGs and has a strong co-occurrence relationship between *aac(6)-Ib-cr, bla*_CTX-M-9_, *bla*_OXA-10_, *bla*_OXA-2_, and *qnrS.* The carbapenemase genes, *bla*_V IM-1_ and *bla*_OXA-58_, also showed a strong and significant correlation (Module 4), whereas, modules 2 and 3 had weaker co-occurrence relationships.

## Discussion

Antibiotic resistance has been extensively studied in human pathogens and in the clinic, however, during the last decade, the focus has been diverted to studies on the reservoirs of ARGs in natural environments impacted by high anthropogenic activity (Khan et al., 2013; Rutgersson et al., 2014; Xiong et al., 2014; Devarajan et al., 2016). Although, it is important to monitor the occurrence of ARGs in highly polluted environments where high selection pressure exerted by antimicrobial contaminants contribute toward the spread and persistence of antibiotic-resistant bacteria, it is critical not to ignore the emergence of ARGs in relatively less polluted environments (Williams, 2001; Baquero et al., 2008; Martinez, 2009). Identifying sources of ARGs and their dissemination in relatively less polluted environments will help form strategies to reduce their spread.

The present study focused on the enrichment and detection of ARGs in culturable Gram-negative bacteria from directionally connected sites; hospital wastewater, WWTP, river, and lake in Sweden, and their possible route of dissemination into the environment. Although high numbers of ARGs were detected in hospital wastewater, there was no evidence that all of these genes were transported downstream to the WWTP. For instance, carbapenemase genes *bla*_IMP-1_*, bla*_IMP-2_, and *bla*_OXA-23_ were present in the hospital, however, absent in all the downstream locations. Presumably, bacteria that harbor these genes are selected for in hospital wastewater and are either diluted by increasing water and/or outcompeted by environmental bacteria due to the fitness cost of carrying ARGs and lack of antibiotic selection pressure in downstream natural environments (Andersson, 2006). The absence of some ARGs in the downstream waters can also be explained by bacterial adaptation to the changing environmental conditions where some bacteria can transform into a non-growing viable but non-culturable (VBNC) state (Colwell, 2000). The VBNC state is an important survival strategy against unfavorable conditions and has been discovered in over 85 bacterial species (Ayrapetyan et al., 2015).

Although wastewater treatment had reduced the number of ARGs and coliforms from raw sewage, as shown in previous studies (Rodriguez-Mozaz et al., 2015), the river receiving treated wastewater still contained a high number of ARGs, similar to the raw wastewater. Moreover, *bla*_OXA-48_, *bla*_CTX-M-8_, and *bla*_SFC-1_ genes were detected only in the downstream river water. Together, these results indicate that WWTP may have introduced the majority of the ARGs into the downstream river. In a separate study, *E. coli* carrying the *bla*_OXA-48_ gene was isolated from the downstream river water in Örebro, which was not previously reported by the clinics in Örebro County (unpublished data), suggesting that the ARGs may be present in the community or there are other sources of contamination for the environment besides hospital wastewater. The upstream river contained few ARGs compared to downstream river sites. This is attributed to low anthropogenic activity in the proximity of the upstream river. Most of the land is dedicated to agriculture with a few animal farms. Since 1986, antibiotics are not used in Sweden as growth promoters in animal husbandry, and only a few antimicrobials are used for treating animal infections such as penicillins, trimethoprim, and sulfonamides (Folkhälsomyndigheten, 2016). Therefore, antibiotic resistance in the upstream environment is expected to be minimal and was half that of the ARG detected in the river downstream of the WWTP. Similar trends were observed in a study performed in Linköping city, Sweden, where a higher abundance of ARGs (*sul1, tetA, tetB*, and *dfrA*) was observed in locations downstream of WWTP (Berglund et al., 2015). Since WWTP appear to be a major source of ARGs, strategies to reduce the environmental impact should focus on reducing the load of ARGs and bacteria in the treated effluents. Studies have shown that advanced treatment (i.e., UV and ozone) of wastewaters prior to release could efficiently reduce the levels of ARGs distributed into the environment (Jäger et al., 2018).

Other sources besides the WWTP effluent that can contribute to the spread of ARGs in the river include migratory birds. Several studies have shown that migratory birds are reservoirs for bacteria harboring *bla*_OXA-48_, and *bla*_CTX-M_ genes and thus provide a source of ARGs contamination in surface waters (Cole et al., 2005; Bouaziz et al., 2017). A Swedish study found that *bla*_CTX-M_ harboring *E. coli* isolated from black-headed gulls were genotypically similar to the clinical *E. coli* isolates from the same city (Bonnedahl et al., 2010). This suggests that ARGs spread in the environment through several routes. In developing countries, effluents from hospitals and WWTP are a major source of human pathogens and ARGs in the environment (Spindler et al., 2012; Marti et al., 2013; Devarajan et al., 2016). In the era of increased international travel and globalization, ARGs are not confined to the countries where they are most prevalent (Sheppard et al., 2016), and can be carried by travelers between countries that can perpetuate the spread in the community (Williams, 2001). A recent study of the gut resistome of 122 travelers demonstrated high acquisition rates of extended spectrum β-lactamase (ESBL) gene *bla*_CTX-M_ after international travel (von Wintersdorff et al., 2014). Upon their return, travelers may spread resistant bacteria in the community, even in countries with a low antibiotic resistance profile, contributing to the high ARG diversity in countries such as Sweden.

Most of the class B β-lactamase (Metallo-β-lactamases) genes found in hospital wastewater were rarely observed in environmental samples. In Sweden, the incidence of community-acquired infections caused by carbapenemase-producing Enterobacteriaceae has been increasing in recent years (SWEDRES-SVARM, 2014). However, the present study showed that the occurrence of carbapenemases in Enterobacteriaceae and other Gram-negative bacteria in the environment is still low and is attributed to *bla*_OXA-48_ and *bla*_V IM_ genes. To best of our knowledge, this is the first study to report *bla*_OXA-48_ in a Swedish river. The OXA-48 is an important carbapenemase enzyme that has been detected in rivers in France (Girlich et al., 2010), Portugal (Poirel et al., 2012), Switzerland (Zurfluh et al., 2013), China (Chen et al., 2010) and United States (Aubron et al., 2005), showing its increasing global distribution. The *bla*_V IM_ genes are commonly detected metallo-β-lactamases and are an emerging worldwide determinant of carbapenem resistance in Gram-negative bacteria (Poirel et al., 2007). The bla_V IM-1_ harboring Enterobacteriaceae have caused several outbreaks in some European countries such as France, Germany, and The Netherlands (Nordmann and Poirel, 2014), while in Greece, VIM-1-producing Enterobacteriaceae are considered endemic (Miriagou et al., 2008; Psichogiou et al., 2008). Furthermore, the *bla*_V IM_ has been detected in rivers in many European countries such as Spain (Piedra-Carrasco et al., 2017), Portugal (Quinteira and Peixe, 2006) and Sweden (Khan et al., 2018), indicating that these important ARGs are not confined to clinical settings (Quinteira and Peixe, 2006; Goren et al., 2010).

Selection of antibiotic-resistant bacteria in a population has been known to occur at high antibiotic concentrations [higher than minimum inhibitory concentration (MIC)]. However, recent studies suggest that resistant bacteria are also selected at sub-inhibitory concentrations, and are more stable in a bacterial population than mutants selected at higher concentrations (Andersson and Hughes, 2010; Sandegren, 2014). Studies have shown that low levels of certain antibiotics enhance the rates of genetic rearrangements and facilitate HGT, thus contributing to the spread of antibiotic resistance (Bahl et al., 2004; Lopez et al., 2007). Co-occurrence network revealed that the groups of ARGs conferring resistance to different antibiotics co-exist in a particular environment, suggesting that multiple ARGs will be co-selected when one gene in a cluster is selected. It is therefore important to formulate strategies targeting these clusters in order to stop the spread of ARGs in the natural environments.

Culture-independent methods such as metagenomics are becoming important tools in understanding the emergence and evolution of antibiotic resistance. However, applying metagenomics for detecting antibiotic resistance in countries with low antibiotic resistance profiles may produce false-negative results as less prevalent bacteria and ARGs are unlikely to be detected. For instance, in a previous study, no carbapenemase genes were detected in a Swedish lake using metagenomics (Bengtsson-Palme et al., 2014). However, it is important to note that culture-dependent methods also have limitations as some environmental bacteria cannot be cultivated with existing methods (Pace, 1997). Furthermore, culture-dependent methods are less likely to detect ARGs carried by VBNC bacterial population or extracellular DNA that may contribute to the spread of ARG by HGT. While culture-independent methods are more suitable for detecting ARGs carried by non-culturable bacteria or extracellular DNA in the environment, the levels of these genes may still be too low for detection in some environments. In the present study, ARGs were detected from the environmental samples using qPCR and enrichment of culturable and clinically relevant Gram-negative bacteria on Chromocult coliform agar. This approach allowed the detection of ARGs that were present in low copy numbers and in clinically relevant bacteria found in these environments.

Taken together, culture-dependent enrichment methods and qPCR can be used for detecting diverse ARGs belonging to class A, B C, D β-lactamase, fluoroquinolones, tetracycline, and aminoglycosides resistance of clinically relevant Gram-negative bacteria from environmental samples with a low antibiotic resistance profile. Hospital wastewater contained a high diversity of ARGs, including the clinically relevant carbapenemase genes, however, low-level presence of these genes in aquatic environments is still a concern. Overall, the present study supports the proposal that WWTP contributes to the dissemination of ARGs in natural environments. Elimination of antibiotic-resistant bacteria and ARGs from the hospital and household wastewaters is an important step to maintain the low-levels of resistance in environmental waters.

## Data Availability

All datasets generated for this study are included in the manuscript and/or the Supplementary Files.

## Ethics Statement

This work deals with environmental and wastewater samples. No clinical or patient samples were collected. The Swedish law does not require ethical permits for work with environmental or wastewater samples.

## Author Contributions

All authors conceived and designed the experiments, analyzed, and interpreted the data. FK and JJ collected the samples. FK performed the experiments and wrote the initial draft of the manuscript. JJ and BS critically revised the manuscript.

## Conflict of Interest Statement

The authors declare that the research was conducted in the absence of any commercial or financial relationships that could be construed as a potential conflict of interest.

## Footnotes

1 www.orebro.se
